# Research ethics in animal models

**DOI:** 10.1590/S1808-86942012000200020

**Published:** 2015-10-20

**Authors:** Ivan Dieb Miziara, Ana Tereza de Matos Magalhães, Maruska d'Aparecida Santos, Érika Ferreira Gomes, Reinaldo Ayer de Oliveira

**Affiliations:** a Associate professor of otorhinolaryngology, São Paulo University Medical School – FMUSP (in charge of the Stomatology and Otorhinolaryngology/AIDS groups of the Otorhinolaryngology Clinical Division, Clinic Hospital, FMUSP); b Speech therapist; master's degree student, FMUSP (speech therapist, member of the Cochlear Implant Team, Clinic Hospital, FMUSP, São Paulo, SP); c Otorhinolaryngologist (doctoral student, Otorhinolaryngology Discipline, FMUSP); d Otorhinolaryngologist and general surgeon; member of the Interdisciplinary Cranial Base Team, Fortaleza General Hospital/SUS (doctoral student of otorhinolaryngology, FMUSP; counselor of the Ceara Regional Medical Board); e Assistant professor of bioethics, Department of Forensic Medicine, Medical Ethics, Social and Work Medicine, FMUSP (counselor of the Sao Paulo Regional Medical Board – CREMESP; coordinator of the Bioethics Technical Chamber, CREMESP). Otorhinolaryngology Discipline, FMUSP. Department of Forensic Medicine, Medical Ethics, and Work Medicine, FMUSP

**Keywords:** animal use alternatives, ethics, ethics committees, research, models, animal

## Abstract

The use of animals in scientific experiments has beendescribed since the fifth century BC. A number of scientific advances in health are attributed to animal models. The issue of the moral status of animals has always been debated.

**Objectives:**

This article aims to review and to present a historical summary of the current laws, to guide researchers who wish to use animal models in otolaryngology research.

**Material and Methods:**

Research on the medline database.

**Results:**

For many years there were no laws ruling the use of animals in scientific experimentation in Brazil. Standards set by national and international organizations were followed. Recently, Law No. 11.794/08 established procedures for the scientific use of animals. Studies in otolaryngology have used the larynxes of rabbits, pigs, dogs, guinea pigs (*Cavia porcellus*), and mice. There were also studies comparing rabbits, rats, and dogs, rhinoplasty on rabbits, and inner ear studies on rats and guinea pigs (albino).

**Conclusions:**

The researchers involved in scientific work with animals should know the principles of Law 11.794/08 and investigate what animals are appropriate for each area of study in their models. Otolaryngologists, especially those dedicated to research, need to be mindful of the ethical rules regarding the use of animals in their studies.

## INTRODUCTION

The first reports of animals being used in scientific experiments date from the fifth century BC. More intensive use of animals in science, however, started in the beginning of the 19^th^ century. Several scientific advances in health have been attributed to animal models.

The issue of the moral status of animal has often been debated; many philosophers thought about this topic, but controversies remain to this day and no consensus has been reached about the status of animals relative to human beings^1^.

There used to be no laws regulating animal use in scientific experiments in Brazil; at that time, guidelines and principles elaborated by domestic and international organizations were applied. Recently, Law n^o^. 11.794/08 (Arouca Law), which defines procedures for using animals in science, was sanctioned^2^.

Several animal species are used in otorhinolaryngology research; each area of this specialty has specific animal models.

The purpose of this paper is to present a historical review and a summary of the current laws to guide researchers that intend to use animal models in their research projects.

## REVIEW OF THE LITERATURE

### Animal experiments

The use and care of animals are mentioned around 500 BC. Pythagoras then believed in metempsychosis; according to this doctrine, a soul may successively animate the bodies of humans, animals, or even plants. Studies in this are probably started with Hippocrates (450 BC), who related the aspect of diseased human organs with those of animals. Alcmaeon (500 BC), Herophilus (330–250 BC), and Erasistratus (305–240 BC) were anatomists that practiced vivisection to observe structures and to formulate hypotheses about their function. Aristotle (384–322 BC) made comparative studies of human and animal organs, and found similarities and differences in their size and function^3^. About 500 years later, Galenus (131–201 AD) became known as one of the fathers of experimental medical science because of his experimental vivisections; Vesalius (1514 to 1564) took up these experiments. In 1638, William Harvey published what was probably the first scientific study involving animals, Exercitatio anatomica de motu cordis et sanguinis in Animalibus. He presented the circulatory physiology of more than 80 animal species. René Réaumur (1683–1757) and Stephen Hales (1677–1761)^4^ made further contributions.

### In defense of animals

Jeremy Bentham (1748–1842), a British philosopher, was historically responsible for systematizing utilitarism, an ethical doctrine that states that an action is correct if it provides intrinsic benefit to society; in other words, the higher the benefit, the better the action is. This benefit should be applied to all beings with sensitivity – it is legitimate to include all animals when defining the morality of an act. He states in his book Introduction to Principles of Morals and Legislation: “...the question is not whether animals can reason, nor can they talk. The true question is this: Can they suffer?” It is likely that the first measures for protecting animals arose based on his ideas.

The first society for the protection of animals was created in England in 1824 – the Society for the Preservation of Cruelty to Animals.

Claude Bernard, the father of contemporary experimental physiology, caused discontent in his wife by experimenting on his daughter's pet dog for demonstration purposes in his classes in 1860; she went on to create the first association for defending laboratory animals.

The first law regulating the use of animals in research was proposed in Great Britain in 1876 – the British Cruelty to Animal Act. The first North-American publication on ethical aspects of experiments in animals was created as late as 1909.

William M.S. Russell and Rex L. Burch (1959) published a book in which they established the principle of the “3 Rs” (Replace, Reduce, and Refine) for research; the intention was to use animals in research by rationalizing the resources and humanizing the care of animals. They argued that animals should be replaced by alternative methods, such as in vitro testing, mathematical models, and computer simulations. An alternative is to reduce the number of animals used in experiments and raising the quality of statistics as applied to small samples. Techniques can be refined to reduce pain and suffering in animal research; this includes care with analgesia and per-, per-, and postoperative antisepsis.

Debates on the use of animals in research and other activities – slaughterhouses, husbandry, transportation, and the cosmetics industry – returned to the scene probably because of professor Peter Singer's book (Animal Liberation) in 1975. It was polemic because it presented reports on the conditions of animals in experiments. It also contributed towards including safeguards for using animals in the 1975 Declaration of Helsinki, which was adopted in the 29^th^ World Medical Assembly in Japan (also in 1975). Animal Liberation, which was edited in Brazil in 2004, has been cited as the most important book on ethics applied to animals. UNESCO announced the Universal Declaration of Animal Rights in a 1978 meeting in Brussels, which brought major topics to the fore^5^.

The opening articles of the Declaration speak of equal rights for the existence of all animals and the right to be respected by humans. Article 8 states that animal experiments that cause physical or psychological suffering are not compatible with animals rights, irrespective of being a medical, scientific, commercial, or other type of experimentation, and that substitution techniques should be developed and used.

During the 1980s the movement to abolish the use of animals in biomedical research grew rapidly, especially in the United States, Britain, Canada, and Australia. The actions by protesters reached such a degree, that the World Medical Association published a specific declaration on the use of animals in research^6^.

### Brazilian law

The first Brazilian law on the rights of animals was the Law n^o^. 24,645 (10 July 1934); it was never revoked neither regulated. It established that all animals were protected by the State and set fines and penalties for whoever mistreated animals. Clause IV of the 3^rd^ paragraph included as definitions of mistreatment “Voluntarily beating, wounding, or mutilating any organ or tissue, except for castration of pets or other operations to benefit animals exclusively, as well as those required for defending humans or those in the interests of science.”

Law n^o^. 6,638 was published in May 1979; it established the guidelines for didactic-scientific practices in animal vivisection. The Law of Environmental Crimes was sanctioned in 1998; it defines the crime and sets fines and penalties for whoever abuses, mistreats, hurts, or mutilates native or exotic wild, domestic, or domesticated animals. The penalties also applied to any painful or cruel experimentation in living animals – even if for didactic or scientific purposes – if there were alternative approaches.

At present, Law n^o^. 11,794/08 (Arouca Law) regulates the clause VII of the 1^st^ paragraph in Article 225 of the Federal Constitution, by establishing procedures for the scientific use of animals. This Law revoked the Law n^o^. 6,638 (8 May 1979). The decree 6,899 – which regulates the Arouca Law – was published on 15 July 2009.

The Arouca Law gave rise to the National Council for the Control of Animal Experimentation (CONCEA) and mandated the creation of Institutional Animal Care and Use Committees (CEUA) at institutions with teaching or research activities using animals. Among other requirements, chapter IV of this law provides instructions about the conditions for raising and using animals for teaching and research purposes, and states that animals may only be submitted to the recommended intervention experimentation protocols in research or learning programs if they are given special treatment – as defined by the CONCEA – before, during, and after any experiment.

If needed, euthanasia should be carried out according to the guidelines of the Ministry of Science and Technology. Whenever possible, teaching sessions should be photographed, filmed, or recorded to be reproduced for illustrating future practices. The number of animals required for any project and the duration of each experiment should be the minimum needed to yield a conclusive result. Adequate anesthesia and analgesia should be used – neuromuscular blockers should not be used in their place.

A specific authorization is required from the CEUA if the study object concerns the pain mechanism. The same animal should not be used again after the main goal of the study is fulfilled. In teaching programs, several traumatic procedures may be carried out in the same animal as long as all procedures are done under the effect of a single anesthetic and that the animal be euthanized before becoming conscious.

Articles 17 and 18 define the penalties for institutions and people that disobey its regulations; this includes reprimands, fines, suspension of activities, and temporary of definitive interdiction of regulated activities.

### Experimentation animals in otorhinolaryngology

Researchers should search the literature to choose the most adequate animals for specific scientific investigation in each area of study^6^, ^7^, ^8^.

There are three main mammal species used in Brazilian laboratories: rats (because of size and quantity), rabbits (calm and easy to handle), and dogs (because of size and anatomical configuration)^6^, ^7^, ^8^. A search on the Bireme website for the words “*animal*” and “*otorhinolaryngology*” for studies published in Portuguese showed the following: studies of the larynx used rabbits, pigs, dogs, guinea-pigs (*Cavia porcellus*), and mice; studies of the face used rabbits, rats, and dogs; studies of rhinoplasty used rabbits; and studies of the inner ear used guinea pig and albinos.

Albuquerque et al.^4^ has commented that the most frequently used animals in research applied to otology are guinea-pigs and rats – their ears are similar to the human ear. These authors concluded that guinea-pigs are better for most procedures, but that rats may be used in some cases^4^.

### Alternatives to using animals

Alternative and in vitro models are being used increasingly in health research.

Brazilian scientists have developed mathematical models of aerodynamic and viscoelastic equations for the larynx; computer software simulates the anatomy and physiology of the larynx, and may be used as teaching tools.^6^ Recently, a Canadian group developed a virtual station for teaching laryngeal anatomy and surgery based on reconstructions of computer tomography and magnetic resonance imaging sections^7^.

Brazilian otorhinolaryngologists have also helped develop a model for simulating nasosinusal endoscopic surgery and procedures of the base of skull – S.I.M.O.N.T. (Sinus Model Otorhino Neuro Trainer) – that simulates a complete surgical procedure with soft tissue consistency and realistic bleeding; it is a useful training tool. The S.I.M.O.N.T. simulates nasal, sinus, and anterior base of skull operations, and it is possible to use it to observe diseased tissue with inflammation, cysts, and tumors.

Software developed in Denmark, which is available for download free of charge – has been developed for studying the ear and for simulating temporal bone dissections. It is called “The visible ear simulator” and is based on high definition images of temporal bones taken from recently-deceased frozen human cadavers. Computers need to have high-definition video cards and an optic pointer to enable interaction when simulating an operation^8^.

Several teaching institutions worldwide have sought to develop research and teaching models to replace animals in experiments.

## FINAL COMMENTS

Protests by anti-vivisectionists against research using animals are currently more frequent.

Studies involving animals require careful planning, knowledge of the laws and guidelines in any given country, ethical principles, rules and conditions from the journal in which the study is to be published; importantly, knowledge about previous studies in the same area is paramount. Researchers should bear in mind the study objectives and be very clear about the need for using live animals.

Animal rights should be respected, as should the development of research for the benefit of humankind. Respect, knowledge, and responsibility should underline any plan for using animals in research^5^.
